# Diagnostic Ultrasound-Based Investigation of Central vs. Peripheral Arterial Changes Consequent to Low-Dose Caffeine Ingestion

**DOI:** 10.3390/nu16020228

**Published:** 2024-01-10

**Authors:** Yu-Bin Jin, Jeong-Hyeon Kim, Chae-Hyeon Song, Chansol Park, Chang-Ki Kang

**Affiliations:** 1 Department of Radiological Science, College of Health Science, Gachon University, Incheon 21936, Republic of Korea; yubin5623@gachon.ac.kr (Y.-B.J.); vchlrkdv@gachon.ac.kr (J.-H.K.); cogus1205@gachon.ac.kr (C.-H.S.); 2 Department of Health Science, Gachon University Graduate School, Incheon 21936, Republic of Korea; 3 Neuroscience Research Institute, Gachon University, Incheon 21565, Republic of Korea

**Keywords:** low-dose caffeine, diagnostic ultrasound, common carotid artery, radial artery, peak systolic velocity, pulse wave velocity, accelerated photoplethysmography

## Abstract

Caffeine is present in various foods and medicines and is highly accessible through various routes, regardless of age. However, most studies on caffeine have focused on the effects of high-dose caffeine ingestion based on the recommended daily amount for adults. In this study, we examined the physiological changes in the central and peripheral vessels that may occur when ingesting low-dose caffeine due to its high accessibility, with the aim of creating an environment of safe caffeine ingestion. This study included 26 healthy participants in their 20s. Peak systolic velocity (PSV), heart rate (HR), and pulse wave velocity (PWV) for vascular stiffness assessment were measured at 0, 30, and 60 min after caffeine ingestion using diagnostic ultrasound to determine the physiological changes in the blood vessels, common carotid artery (CCA) and radial artery (RA). In addition, percutaneous oxygen saturation (SpO_2_), blood pressure (BP), and accelerated photoplethysmography (APG) were measured. In comparison with before ingestion, the HR tended to decrease and showed a significant difference at 30 and 60 min (*p* = 0.014 and *p* = 0.031, respectively). PSV significantly decreased in both vessels at 30 and 60 min (*p* < 0.001 and *p* < 0.001, respectively). APG showed a decreasing trend until 60 min after ingestion, with a significant difference at 30 and 60 min (*p* = 0.003 and *p* = 0.012, respectively). No significant difference was observed in SpO_2_, BP, or PWV; however, they showed a tendency to increase after ingestion. Decreased HR may occur because of the baroreflex caused by an increase in BP. The RA has many branches and a smaller diameter; therefore, the PSV was lower in the RA than that in the CCA. This effect can occur because of the difficulty in the smooth expansion of blood vessels, which leads to a decrease in blood flow. In addition, an increase in intracellular calcium concentration can prevent vasodilation and increase the propagation velocity of pulse waves. The reflected waves can increase systolic blood pressure but reduce PWV and vascular elasticity. These results suggest that even low-dose caffeine can improve blood vessel health by providing temporary stimulation to the blood vessels; however, it can also cause changes in blood flow and blood vessel elasticity, which can lead to serious diseases such as stroke and high blood pressure. Therefore, caution should be exercised when caffeine consumption is indiscriminate.

## 1. Introduction

Caffeine is an ingredient in various foods and medicines, and caffeine consumption has continued to increase over the past 20 years, with approximately 80% of the world’s population ingesting products containing caffeine [1,2]. Caffeine induces cognitive stimulation and exerts prolonged physiological effects in the body after ingestion, with a half-life of 2.5 to 10 h [3]. Caffeine belongs to the xanthine family and is known to block the binding of adenosine to receptors [4]. As a neuromodulator that plays various roles in the body, adenosine has a direct impact on heart and vascular function. The binding of adenosine to adenosine 2A (A_2A_) receptors induces blood vessel dilation and regulates blood flow [5,6]. However, when caffeine enters the blood vessels, it binds to the A_2A_ receptors in vascular smooth muscle cells and blocks adenosine binding, preventing blood vessel dilation and causing continued contraction [7,8]. These changes affect central blood vessels, such as the common carotid artery (CCA), which supplies blood to the brain, rather than peripheral blood vessels, such as the radial artery (RA). It also has an important impact on the diagnosis and treatment of vascular diseases such as angina pectoris [9]. Large arteries, such as the CCA, perform the main function of the cardiovascular system by absorbing part of the ventricular separation energy during systole and releasing it during diastole, maintaining blood flow similar to the coronary arteries and promoting blood flow to the periphery [10]. However, caffeine consumption causes vascular stiffening, increasing both pulse wave velocity (PWV) and reflected wave amplitude, which increases the central systolic blood pressure [10]. This phenomenon may increase systolic and pulse pressure and reduce coronary blood flow [11]. This increased stiffness of large arteries is associated with a variety of cardiovascular risk factors, including hypertension, diabetes, and atherosclerosis [12,13]. Moreover, caffeine exerts pharmacologically active responses that stimulate the nervous system, decreasing norepinephrine release in sympathetic nerve endings, which induces vasodilation [14], and stimulating the secretion of serotonin and circulatory catecholamines from the cerebral cortex, which improves the action of the sympathetic nervous system [3]. An increased circulatory catecholamine level causes vasoconstriction, thereby increasing blood pressure (BP) [3]. Caffeine also helps prevent kidney stone disease by reducing ureteral reabsorption in the kidneys, promoting renal vasodilation, and increasing urine production and glomerular blood flow, filtration rate, and output [15]. Consuming 300 mg of caffeine daily has been found to increase energy expenditure by approximately 79 kcal/day, indicating that caffeine ingestion can help regulate body weight [16].

However, research on the side effects of caffeine ingestion has revealed that continuous or excessive consumption of caffeine causes anxiety and dehydration, and in the case of addiction, it can cause nervousness, muscle spasms, and sleep disorders. Increased BP because of increased vascular resistance and cardiac output can cause hypertension [10,17,18]. Caffeine ingestion impedes sleep even 6 h after ingestion, thereby causing sleep disorders and reducing the quality of sleep [19]. In addition, the pulse rate, arterial stiffness, and vascular resistance increase after caffeine ingestion, causing acute compression owing to vasoconstriction, which increases BP; the plasma concentration also increases 1 h after ingestion [18,20]. Systolic BP (SBP) in the central blood vessels increases more markedly than that in the peripheral blood vessels after caffeine ingestion [14]. The rapid ingestion of 300 mg of caffeine by a healthy adult can affect cardiovascular function [20], and changes in blood vessels can cause side effects such as high BP, heart failure, arrhythmia, and cerebrovascular diseases such as stroke [10]. Therefore, patients with cardiovascular disease, including those with hypertension, should be cautious about caffeine ingestion.

A typical source of caffeine is coffee, and with the recent increase in coffee consumption, caffeine ingestion has also increased [21]. Accordingly, each country recommends an appropriate amount of caffeine—in 2017, the European Food Safety Authority (EFSA) in Europe set the recommended caffeine ingestion dose at 400 mg/day for adults, 200 mg/day for pregnant women, and 3 mg/kg/day for children [22]. The United States Food and Drug Administration (U.S. FDA) reported an average caffeine ingestion of 280 mg/day through coffee and caffeine-containing foods. The Ministry of Food and Drug Safety of the Republic of Korea recommended a maximum daily caffeine ingestion for adults of <400 mg and reported that adults ingest an average of 81.9 mg of caffeine per day [23,24]. Although caffeine is mostly ingested via coffee, it is also included in various foods and drinks such as chocolate, cola, and green tea, as well as in medicine. For example, a cup of roasted coffee contains about 85 mg of caffeine, green tea has 20 to 30 mg, and cola has 18 mg [25]. Therefore, even if coffee is not consumed, caffeine may be ingested through other sources. This makes it difficult to calculate the amount of caffeine ingested, making indiscriminate exposure to caffeine possible [26]. There is a lack of caution regarding neurological and cardiovascular side effects, such as overdose and intoxication, that can occur with indiscreet intake to obtain cognitive stimulant effects, which are the reported benefits of caffeine ingestion. Caffeine causes nutrients such as calcium and vitamins to be excreted from the body and prevents their absorption; therefore, care must be exerted when consuming coffee during adolescence because of the risk of growth decline or disease [27].

Coffee causes various physiological changes in the body, and owing to increased coffee consumption, in-depth research on the physiological changes and vascular effects of caffeine ingestion is needed. Previous studies have dealt with the ingestion of a high-dose caffeine, averaging more than 200 mg, which is higher than the caffeine content in a cup of coffee and close to the recommended daily ingestion for adults. However, caffeine is found in a variety of foods and medicines other than coffee, and a low-dose caffeine can be ingested even without consuming coffee. Coffee has high accessibility through various routes regardless of age. Hence, identifying the occurrence of physiological changes that accompany low-dose caffeine ingestion rather than the effects of high-dose caffeine ingestion (>200 mg) and examining the differences between the post-ingestion responses of the central and peripheral blood vessels with different caffeine sensitivities is essential.

Accordingly, we aimed to examine the physiological changes in the central and peripheral blood vessels over time when a low-dose caffeine was consumed using diagnostic ultrasound and patient monitoring devices to provide general information on the physiological changes caused by consuming low-dose caffeine and help create an environment of safe caffeine consumption.

## 2. Materials and Methods

### 2.1. Participants

This study and its research procedures were approved by the Institutional Review Board (IRB). Recruitment for this study was conducted with an advertisement. A total of 26 healthy participants (mean age: 22.08 ± 1.51 years, mean ± standard deviation; 13 males and 13 females) voluntarily enrolled in this study. The average daily caffeine ingestion by the participants was approximately 111.88 mg ± 92.88. Before the experiment, a consent form for participating in this study was obtained after explaining the purpose of this study, the research procedures, and the safety of the equipment sufficiently to the participants.

The inclusion criteria were healthy adults ≥ 20 years old who had no adverse effects due to caffeine and did not have cerebral or cardiovascular diseases. Patients taking cardiovascular drugs and those with diseases of the cardiovascular system were excluded. Those with an aversion to caffeine ingestion or side effects, mental or physical weakness, or mental illnesses were also excluded from the experiment.

### 2.2. Experimental Protocols and Data Acquisition

**Figure 1:**
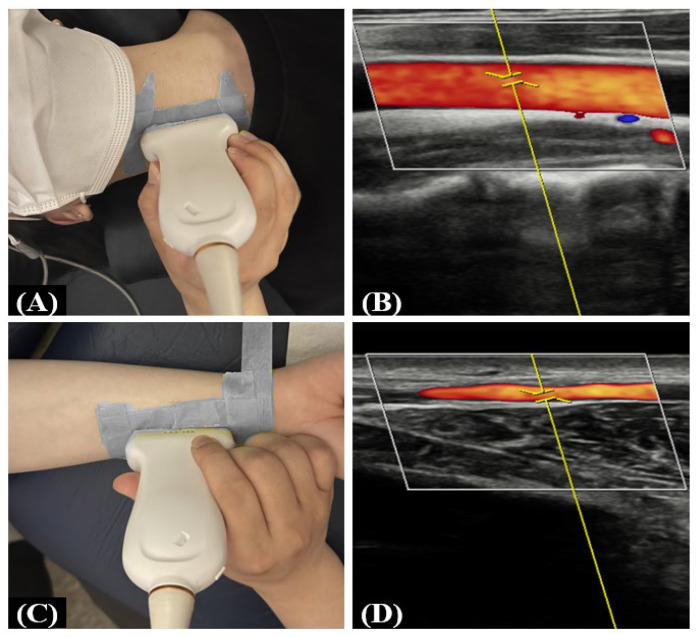
Probe positions for the CCA and RA and their Doppler images. (**A**,**C**) Probe positions for the CCA and RA, respectively. (**B**,**D**) Doppler images of the CCA and RA, respectively. The yellow line represents the angle of the Doppler effect in the Doppler mode of diagnostic ultrasound. The red and blue colors in the Doppler image represent blood flow into and out of the probe, respectively.

To prevent factors other than caffeine from influencing the results of the experiment, before the start of the experiment, the participants stopped smoking and fasted for 6 h, refrained from ingesting caffeine for 12 h, and abstained from alcohol drinking for 24 h, and they were instructed to visit after sufficient rest. After confirming the locations of the right CCA and RA using a diagnostic ultrasound (RS85, Samsung Medison, Seoul, Republic of Korea) and a linear transducer array (LA2-14A, Samsung Medison, Seoul, Republic of Korea) with a frequency bandwidth of 2.0–14.0 MHz. The ultrasound measurement was performed by keeping the participant’s arm still to limit movement, and the positions of the probe were marked for repeated measures (Figure 1).

Participants consumed 100 mL of coffee containing approximately 100 mg of caffeine. Considering that it takes approximately 45 min for 99% of caffeine to be absorbed from the blood, physiological changes and vital signs in the blood vessels were measured three times: before ingestion (0 min) and 30 and 60 min after caffeine ingestion [28].

To examine the physiological changes in the blood vessels because of caffeine ingestion, the peak systolic velocity (PSV) of the CCA and RA and the heart rate (HR) were measured using ultrasound color Doppler mode (C-mode). The maximum and minimum diameters (MaxD and MinD, respectively) of each vessel were measured to derive the stiffness index (SI) and PWV for the evaluation of vascular stiffness. Each measurement was performed on the longitudinal scan images of the CCA and RA for 30 s. In addition, a smartwatch (SM-R850, Samsung Electronics, Suwon, Republic of Korea) was used to measure the SBP and diastolic BP (DBP), which are required for calculating the SI and PWV, along with the MaxD and MinD after calibration was performed for each participant before the experiment. To measure the oxygen consumption due to caffeine ingestion, percutaneous oxygen saturation (SpO_2_) was measured for 30 s using a sensor on a patient monitor (Patient monitor, Bionics, Chuncheon, Republic of Korea) placed on the left index finger. To compare the elasticity and stiffness of the blood vessels, a sensor for autonomic nervous system activity (SA-3000new; MEDICORE Co., Seongnam, Republic of Korea) was placed on the right index finger to measure the accelerated photoplethysmography (APG) for 1 min.

The acquired ultrasound images of the CCA and RA were stored in DICOM format. DICOM Viewer software (Radiant DICOM Viewer Version 2021.2.2 (64-bit), Medixant, Poznan, Poland) was used to record and measure the HR, PSV, MaxD, and MinD of the CCA and RA. The PSV and HR were measured at 0, 30, and 60 min. The MaxD and MinD were measured based on the inner membrane of the blood vessels at the largest and smallest diameters. Images for measurements were extracted from the recorded data at 1-second intervals. After calculating the SI using Equation (1), PWV was calculated using Equation (2).

(1) $$\mathrm{Stiffness}\;\mathrm{index}\;\left(SI\right)=ln\left(\frac{SBP}{DBP}\right)\times \left(\frac{MinD}{∆D}\right),\;where\;∆D=MaxD-MinD$$

(2) $$\mathrm{Pulse}\;\mathrm{wave}\;\mathrm{velocity}\;(PWV)=\sqrt{\frac{SI\times DBP}{2\times BD}}$$

where blood density (BD) = 1.050 g/cm^3^.

### 2.3. Statistical Analysis

Statistical analysis of the experimental results obtained in this study before and after caffeine ingestion was performed with repeated-measures analysis of variance (RM ANOVA) using a statistical program (Jamovi version 2.2.5, free software, https://www.jamovi.org, accessed on 4 December 2023). Changes in vital signs, blood vessels, and blood flow over time (0, 30, and 60 min) were compared. In the RM ANOVA for dependent variables such as SpO_2_, HR, PSV, PWV, and APG, a sphericity test was performed to test the covariances between each time point. In cases where sphericity was not satisfied, the Greenhouse–Geisser correction results were applied. In the RM ANOVA, Tukey’s post hoc test was performed when statistical significance (*p* < 0.05) was satisfied. Differences between genders in physiological values measured after caffeine ingestion were compared using an independent samples *t*-test after a normality test. Statistical significance was set at *p* = 0.05.

## 3. Results

**Figure 2:**
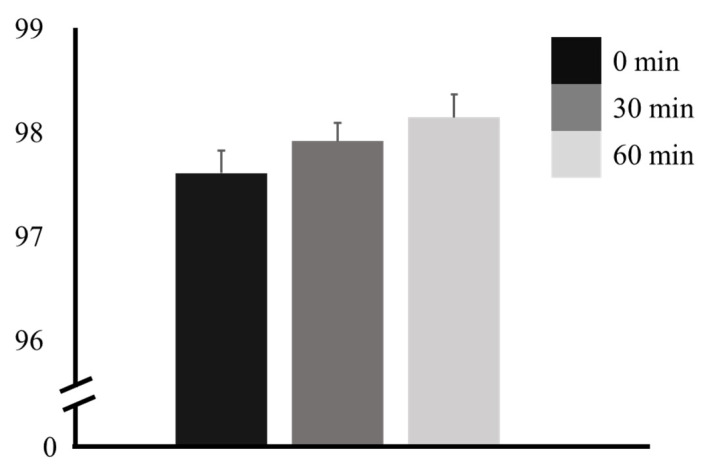
Percutaneous oxygen saturation before and after caffeine ingestion.

SpO_2_ was 97.62 ± 1.10% at 0 min, increased to 97.92 ± 0.89% at 30 min, and further increased to 98.15 ± 1.12% at 60 min. A tendency for an increase at 30 and 60 min after ingestion was observed. However, there was no significant difference (*p* = 0.121) (Table 1 and Figure 2).

**Figure 3:**
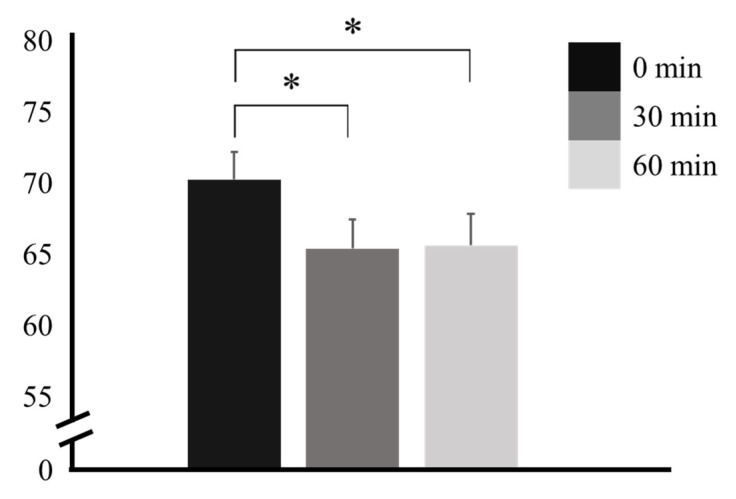
Heart rate before and after caffeine ingestion. * *p* < 0.05.

The HR was 70.31 ± 10.02 bpm at 0 min, abruptly decreased to 65.46 ± 10.36 bpm at 30 min, and remained stable at 65.69 ± 11.21 bpm at 60 min. The changes at 30 and 60 min were significant when compared to the change at 0 min (*p* = 0.014 and *p* = 0.031, respectively). No significant difference was observed between 30 min and 60 min (*p* = 0.984) (Table 2 and Figure 3).

The PSV was 91.45 ± 20.08 cm/s at 0 min, 72.74 ± 16.44 cm/s at 30 min, and 70.42 ± 14.25 cm/s at 60 min in the CCA. There was a significant difference at both 30 and 60 min compared with that at 0 min (*p* < 0.001 and *p* < 0.001, respectively), but no significant difference was observed between those at 30 and 60 min (*p* = 0.544). In other words, the cerebral blood flow (CBF) rate significantly decreased until 30 min after ingestion, with little change between 30 and 60 min.

**Figure 4:**
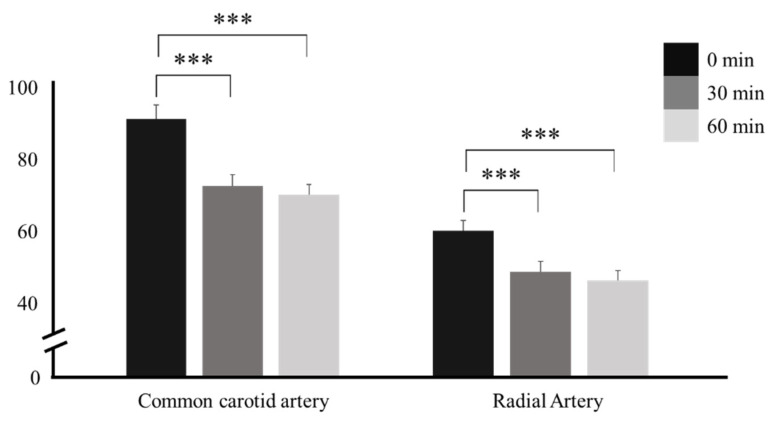
Peak systolic velocity in the CCA and RA before and after caffeine ingestion. *** *p* < 0.001.

In the RA, the PSV was 59.50 ± 14.86 cm/s at 0 min, 48.57 ± 14.25 cm/s at 30 min, and 46.17 ± 13.49 cm/s at 60 min. Significant differences were also observed at 30 and 60 min compared with that at 0 min (*p* < 0.001 and *p* < 0.001, respectively) but not between those at 30 and 60 min (*p* = 0.412). The blood flow rate in peripheral blood vessels, such as the RA, tended to decrease significantly at 30 and 60 min after ingestion compared with that at 0 min. This decrease lasted up to 60 min but was not significant when compared to that at 30 min (Table 3 and Figure 4).

**Figure 5:**
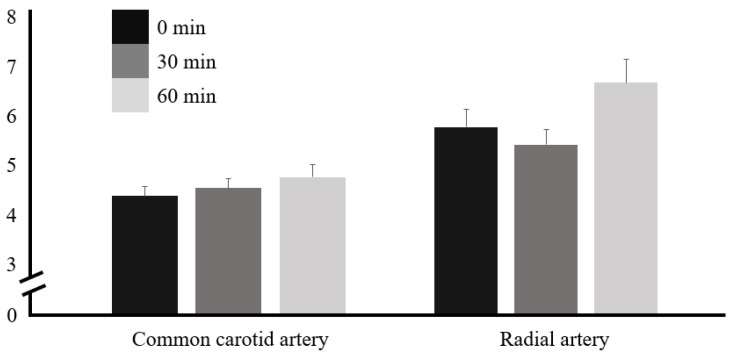
Pulse wave velocity in the CCA and RA before and after caffeine ingestion.

The PWV was 4.39 ± 0.98 m/s at 0 min; it increased to 4.56 ± 0.93 m/s at 30 min and further increased to 4.77 ± 1.31 m/s at 60 min in the CCA, showing a tendency to increase continuously until 30 and 60 min after caffeine ingestion. In the RA, the PWV was 5.69 ± 1.77 m/s at 0 min, 5.34 ± 1.55 m/s at 30 min, and 6.56 ± 2.40 m/s at 60 min, which decreased until 30 min after caffeine ingestion and then tended to increase. However, the PWV in both the CCA and RA had no significant difference before and after caffeine ingestion (Table 4 and Figure 5).

**Figure 6:**
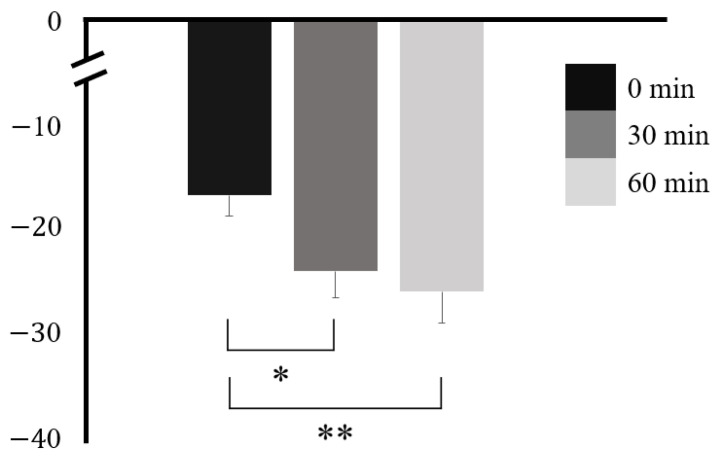
Accelerated photoplethysmography before and after caffeine ingestion. * *p* < 0.05, ** *p* < 0.01.

APG was −16.74 ± 10.24 at 0 min, −24.04 ± 12.97 at 30 min, and −26.04 ± 15.49 at 60 min. There was a statistically significant difference at 30 and 60 min when compared with that at 0 min (*p* = 0.003 and *p* = 0.012, respectively) but not between 30 and 60 min (*p* = 0.554) (Table 5 and Figure 6). APG showed the same tendency as the PSV, with a strong reduction up to 30 min after ingestion and a gradual decline, but a slight reduction up to 60 min.

The SBP in the CCA was 120.68 ± 7.29 mmHg at 0 min, 121.88 ± 7.45 mmHg at 30 min, and 123.00 ± 7.39 mmHg at 60 min, showing a continuous increase after caffeine ingestion. The DBP in the CCA was 71.69 ± 9.68 mmHg at 0 min, 71.46 ± 8.13 mmHg at 30 min, and 72.04 ± 8.91 mmHg at 60 min; however, both the SBP and DBP in the CCA had no significant difference before and after caffeine ingestion (Supplementary Table S1). The SBP in the RA was 120.60 ± 6.92 mmHg at 0 min, 118.48 ± 2.96 mmHg at 30 min, and 122.28 ± 6.06 mmHg at 60 min, which decreased until 30 min after caffeine ingestion and then tended to increase. The DBP in the RA was 71.42 ± 9.60 mmHg at 0 min, 72.12 ± 9.66 mmHg at 30 min, and 72.58 ± 8.08 mmHg at 60 min; however, both the SBP and DBP in the RA had no significant difference before and after caffeine ingestion (Supplementary Table S1).

As a result of comparing the differences in physiological values measured after caffeine ingestion according to gender, it was found that there was no significant difference in physiological changes. At 30 min, SpO_2_ was 97.85 ± 0.18% and 98.00 ± 0.18% in men and women (*p* = 0.937), respectively. The HR was 61.77 ± 1.62 bpm and 69.15 ± 2.20 bpm (*p* = 0.313); the PSV was 77.75 ± 3.66 cm/s and 67.73 ± 2.49 cm/s (*p* = 0.313) for the CCA and 52.79 ± 2.86 cm/s and 44.35 ± 2.58 cm/s (*p* = 0.313) for the RA; the PWV was 4.72 ± 0.22 m/s and 4.39 ± 0.13 m/s (*p* = 0.655) for the CCA and 5.42 ± 0.33 m/s and 5.27 ± 0.20 m/s for the RA; and APG was −23.62 ± 3.29 and −24.47 ± 1.63 (*p* = 1.000), respectively. At 60 min, SpO_2_ was 97.77 ± 0.20% and 98.54 ± 0.22% in men and women (*p* = 0.313), respectively. The HR was 62.54 ± 1.43 bpm and 68.85 ± 2.68 bpm (*p* = 0.334); the PSV was 74.81 ± 2.93 cm/s and 66.02 ± 2.47 cm/s for the CCA (*p* = 0.313) and 51.91 ± 2.68 cm/s and 40.42 ± 2.16 cm/s for the RA (*p* = 0.284); the PWV was 4.93 ± 0.29 m/s and 4.62 ± 0.23 m/s for the CCA (*p* = 0.825), and 6.56 ± 0.47 m/s and 7.00 ± 0.41 m/s for the RA (*p* = 0.655); and APG was −25.88 ± 3.83 and −26.19 ± 2.13 (*p* = 1.000), respectively.

## 4. Discussion

This study aimed to examine the physiological changes in central and peripheral blood vessels over time after low-dose caffeine consumption. We compared the elasticity and stiffness of the central and peripheral blood vessels and autonomic nervous system activity after low-dose caffeine ingestion by analyzing the differences in PSV, PWV, HR, SpO_2_, and APG measured using noninvasive Doppler ultrasound, patient monitoring, and smart devices before and 30 and 60 min after ingestion.

In our study, SpO_2_ showed no significant difference at any time point but tended to increase continuously from 0 to 30 min. This can be attributed to an increase in tissue blood flow caused directly or indirectly by blood vessel dilation, which is attributed to the increase in blood adenosine as a result of the blockade of adenosine receptors following caffeine ingestion [29,30,31]. This appears to be because caffeine delays the recovery of the vagus nerve, which affects oxygen consumption, consequently decreasing oxygen demand and increasing oxygen supply [29,30,31]. However, as shown in the results, no significant differences were observed in the effects of low-dose caffeine ingestion. Moreover, changes in blood oxygen saturation may occur when high-dose caffeine is consumed [32].

There was a significant difference in the HR at 30 and 60 min compared with that at 0 min, but no significant difference was observed between those at 30 and 60 min. The HR decreased significantly up to 30 min after caffeine ingestion. Subsequently, only a slight increase was observed up to 60 min. The decreased HR can be attributed to the lowering heart rate caused by baroreflex activity in response to the increase in BP caused by caffeine ingestion [33,34,35]. These results are consistent with those of previous studies showing that post-exercise caffeine ingestion lowers the HR quickly. This finding indicates that even ingesting low-dose caffeine has a significant effect on HR reduction [31].

A significant difference was observed in the PSV at 30 and 60 min in the CCA and RA compared with that at 0 min, despite low-dose caffeine ingestion; however, no significant difference was observed between 30 and 60 min. In the RA, an abrupt and significant decrease was observed in the PSV between 0 and 30 min, and a gradual but not significant decrease was observed between 30 and 60 min. The rate of change was greater in the central vessel CCA than in the peripheral vessel RA. This effect seems to be influenced by the diameter and blood flow, which decrease depending on the branching of blood vessels from the center to the periphery. Furthermore, this effect seems to be related to the fact that caffeine, when ingested, interacts with adenosine as a non-selective receptor antagonist, binds to adenosine receptors, and prevents vasodilation. This mechanism impedes the smooth expansion of blood vessels, causing them to remain in a contracted state, which decreases the blood flow and PSV [36,37]. These results are consistent with those of previous studies showing decreased PSV in the anterior cerebral artery (ACA) after caffeine ingestion [36]. The CCA measured in this study is a basal vessel that supplies blood to the ACA through the internal carotid artery. Therefore, it is clear that low-dose caffeine ingestion also affects cerebrovascular vessels.

No significant within-subject effects were observed for the PWV in the CCA (*p* = 0.323); however, significant within-subject effects were observed in the RA (*p* = 0.036). Tukey’s post hoc test revealed that there were no temporal changes before or after ingestion. However, a constant increase from 30 to 60 min in the PWV was observed in the CCA, whereas a decrease in the PWV at 30 min and then an increase at 60 min was observed in the RA. This increase showed a greater and later change in the peripheral vessel compared with the central vessel, suggesting a relationship between the composition of the blood vessel wall, that is, peripheral blood vessels are more sensitive to PWV changes than central ones because they are mainly composed of collagen fibers and smooth muscle cells, while central blood vessels respond flexibly to PWV changes as they are mainly composed of elastin [38]. In addition, peripheral blood vessels had a higher PWV than central blood vessels in younger people, and the PWV in central blood vessels increased more than that in peripheral blood vessels in elderly people [38], which is consistent with the higher PWV observed in the peripheral blood vessels shown in this study, wherein the participants’ ages spanned the twenties, reflecting good health status. Furthermore, an increase in intracellular calcium concentration by caffeine ingestion causes smooth muscle cell contraction, which accelerates the propagation of pulse waves due to rigid blood vessels. At this time, the reflected wave increases the SBP and consequently, the PWV [10,39,40]. The increase in PWV observed in this study is consistent with previous research [10]. However, the aforementioned study differs from the present study in that it investigated the effects of ingesting 150 mg of caffeine, and PWV was measured using the foot-to-foot method. In the foot-to-foot method, PWV is measured over a wide range of body parts, which is different from this study, where the PWV of a specific blood vessel was measured using a one-point measurement method. In this study, the participants ingested 100 mg of caffeine, which is a relatively low amount; therefore, no significant difference was observed. However, the results of this study also showed a tendency for the PWV to increase; therefore, even ingesting small amounts of caffeine may cause changes in the PWV in both central and peripheral blood vessels, which may also lead to the development of cardiovascular disease and/or damage to the end organs [41].

The APG at 30 and 60 min were significantly different from that at 0 min; however, no significant difference was observed between 30 and 60 min. After rapidly decreasing from 0 to 30 min, it tended to remain constant between 30 and 60 min. Among the effects of caffeine, the amplitude of reflected waves increased by sympathetic stimulation increases SBP and pulse velocity, leading to an increase in the arterial pulsatile movement of blood vessels and a decrease in the elastic capacity [10,42]. This APG reduction showed the same trend as observed in a previous study [42]. This suggests that even low-dose caffeine ingestion can decrease the elastic capacity of blood vessels, thereby affecting vascular stiffness and increasing PWV. Furthermore, the comparison of gender differences after caffeine ingestion showed no significant differences in all physiological variables, suggesting that low-dose caffeine may cause the same physiological responses regardless of gender.

In this study, to examine the physiological changes associated with caffeine ingestion in the central and peripheral blood vessels, we measured and compared PSV, HR, SpO_2_, PWV, and APG at different time points. The results showed that HR and APG decreased after caffeine ingestion, and PSV decreased in both the CCA and RA. In contrast, PWV increased in both the CCA and RA, and SpO_2_ also increased after caffeine ingestion. In particular, the results showed a rapid change at 30 min and a continuous trend until 60 min. This is consistent with studies showing that caffeine has a half-life of 2.5–10 h after ingestion [3] and induces long-term physiological effects in the body, such as sleep disruption for 6 h after ingestion [19]. However, according to the results of this study, which showed a rapid change 30 min after ingestion, consuming an appropriate dose 30 min before a situation requiring arousal or attention is more efficient, especially in daily life. Furthermore, decreases in HR and APG may be closely related to autonomic function or the prognosis of arteriosclerosis [43,44]. A rapid decrease in APG causes an increase in PWV and a decrease in PSV, which may provide a temporary stress stimulus to the blood vessels and improve vascular health. A previous study examined changes in retinal blood vessels by ingesting 200 mg of caffeine and found that blood vessel diameter decreased and blink response tended to increase, indicating the vasoconstrictive effect of caffeine and an increase in blood pressure due to the autoregulatory response of retinal blood vessels [45]. Additionally, another transcranial Doppler study showed a 22% decrease in CBF and a 13% decrease in middle cerebral artery blood velocity due to a reduction in the middle cerebral artery diameter following 250 mg caffeine ingestion [46]. However, changes in blood vessels caused by caffeine ingestion can reduce blood flow and change the elasticity of blood vessels, leading to serious diseases, such as stroke, hypertension, and heart failure. As the incidence of side effects increases, the mortality rate may consequently increase [47]. Furthermore, caffeine consumption can cause palpitations and increase aortic stiffness, which can impair left ventricular function, reduce coronary blood flow, and cause ischemic heart disease [10,48]. The vasoconstrictive properties of acutely administered caffeine may result in an overall reduction in CBF [49]. Since brain cell metabolism consists of the supply of oxygen and other substances through CBF, a decrease in CBF can cause functional and structural damage to the brain [50]. Insufficient CBF supply can not only cause acute ischemic stroke but also damage neurons and glial cells [51]. Therefore, it is important to be aware of the effects of indiscriminate consumption of caffeine and pay attention to them.

This study has some limitations. First, the study sample size was small and included only young and healthy participants. Further studies should include participants of various ages or those at high risk of developing vascular diseases. Second, this study used commercially available coffee; therefore, ingredients other than caffeine may have affected the results. However, most caffeine is ingested through coffee, suggesting that caffeine ingestion can be replicated by coffee ingestion and the resulting vascular changes caused by caffeine ingestion can be verified using coffee. Third, in this study, physiological variables were measured at 30 min intervals; therefore, studies in which the measurements are taken at shorter time intervals and over a longer period of time are required. Additionally, the results of this study using low-dose caffeine (100 mg) should be compared with those from studies using high-dose caffeine, and the effect on central and peripheral blood vessels requires further study. Further investigation still needs to be performed to determine various effects in the broader context of caffeine research. Various control groups, such as classifications based on caffeine sensitivity and/or daily consumption, should be further considered to reach a concrete conclusion. Additionally, further studies should include participants of different ages and genders or those at high risk of developing vascular disease.

## 5. Conclusions

Caffeine is found not only in coffee but also in various foods and medications. Many studies have been conducted on the effects of coffee that contains a high caffeine content on adults—the main consumers. However, studies on adolescents’ access to caffeinated beverages and caffeine ingestion through foods other than coffee are lacking. The present study examined physiological changes in the central and peripheral blood vessels caused by ingesting low-dose caffeine in young adults and found that SpO_2_, HR, and APG tended to decrease after caffeine ingestion. PSV tended to decrease in the CCA and RA, but PWV tended to increase in the CCA and RA. These results demonstrate that various physiological changes occur in the central and peripheral blood vessels, even with low-dose caffeine ingestion, providing an opportunity to consider the risks of indiscriminate caffeine consumption. Based on the results of this study, establishing a safe caffeine consumption environment using various approaches, including monitoring and formulating an acceptable amount of caffeine that can be ingested considering the age of consumption, is necessary.

## Figures and Tables

**Table 1:** Comparison of percutaneous oxygen saturation (SpO_2_) using RM ANOVA.

Time	F	*p*	Factor 1	Factor 2	Mean Difference ± SE (Factor 1 − Factor 2)	t	*p_Tukey_*
0 min	2.40	0.121	0 min	30 min	−0.308 ± 0.227	−1.35	0.379
30 min				60 min	−0.538 ± 0.538	−1.71	0.221
60 min			30 min	60 min	−0.231 ± 0.213	−1.30	0.411

Abbreviations: F, F statistic; SE, standard error; t, t statistic.

**Table 2:** Comparison of the heart rate (HR) using RM ANOVA.

Time	F	*p*	Factor 1	Factor 2	Mean Difference ± SE (Factor 1 − Factor 2)	t	*p_Tukey_*
0 min	6.19	0.004 *	0 min	30 min	4.846 ± 1.58	3.068	0.014 *
30 min				60 min	4.615 ± 1.70	2.714	0.031 *
60 min			30 min	60 min	−0.231 ± 1.36	−0.169	0.984

Abbreviations: F, F statistic; SE, standard error; t, t statistic. * *p* < 0.05.

**Table 3:** Comparison of peak systolic velocity (PSV) using RM ANOVA.

Vessel	Time	F	*p*	Factor 1	Factor 2	Mean Difference ± SE (Factor 1 − Factor 2)	t	*p_Tukey_*
CCA	0 min	37.5	<0.001 *	0 min	30 min	18.70 ± 2.64	7.09	<0.001 *
	30 min				60 min	21.03 ± 3.09	6.81	<0.001 *
	60 min			30 min	60 min	2.32 ± 2.18	1.06	0.544
RA	0 min	21.9	<0.001 *	0 min	30 min	10.93 ± 2.47	4.42	<0.001 *
	30 min				60 min	13.33 ± 2.06	6.46	<0.001 *
	60 min			30 min	60 min	2.40 ± 1.86	−1.30	0.412

Abbreviations: CCA, common carotid artery; F, F statistic; RA, radial artery; SE, standard error; t, t statistic. * *p* < 0.05.

**Table 4:** Comparison of pulse wave velocity (PWV) using RM ANOVA.

Vessel	Time	F	*p*	Factor 1	Factor 2	Mean Difference ± SE (Factor 2 − Factor 1)	t	*p_nc_*	*p_Tukey_*
CCA	0 min	1.16	0.323	0 min	30 min	−0.166 ± 0.200	−0.832	0.413	0.687
	30 min				60 min	−0.381 ± 0.235	−1.619	0.118	0.256
	60 min			30 min	60 min	−0.215 ± 0.307	−0.700	0.490	0.766
RA	0 min	3.55	0.036 *	0 min	30 min	0.347 ± 0.431	0.805	0.428	0.703
	30 min				60 min	−0.864 ± 0.439	−1.970	0.060	0.140
	60 min			30 min	60 min	−1.212 ± 0.529	−2.291	0.031 *	0.076

Abbreviations: CCA, common carotid artery; F, F statistic; nc, no correction; RA, radial artery; SE, standard error; t, t statistic. * *p* < 0.05.

**Table 5:** Comparison of accelerated photoplethysmography (APG) using RM ANOVA.

Time	F	*p*	Factor 1	Factor 2	Mean Difference ± SE (Factor 2 − Factor 1)	t	*p_Tukey_*
0 min	8.81	<0.001 *	0 min	30 min	7.30 ± 1.94	3.76	0.003 *
30 min				60 min	9.29 ± 2.99	3.11	0.012 *
60 min			30 min	60 min	1.99 ± 1.90	1.05	0.554

Abbreviations: F, F statistic; SE, standard error; t, t statistic. * *p* < 0.05.

## Data Availability

The data presented in this study are available upon request from the corresponding author.

## Supplementary Materials

The following supporting information can be downloaded at: https://www.mdpi.com/article/10.3390/nu16020228/s1. Table S1: Comparison of blood pressure (BP) using RM ANOVA.
