# Supplementary material for: Diagnostic Ultrasound-Based Investigation of Central vs. Peripheral Arterial Changes Consequent to Low-Dose Caffeine Ingestion
Source: Nutrients. 2024 Jan 10;16(2):228. doi: 10.3390/nu16020228 (PMC10820579; doi:10.3390/nu16020228)
Supplement: Supplementary file 1 [file nutrients-16-00228-s001.zip › nutrients-2784227-supplementary.pdf]

Supplementary Table S1. Comparison of blood pressure (BP) using RM ANOVA.

Vessel	BP	Time	F	*p*	Factor 1	Factor 2	Mean Difference ± SE (Factor 1 − Factor 2)	t	p_{Tukey}
CCA	SBP	0 min	1.23	0.303	0 min	30 min	−0.913 ± 1.08	−0.844	0.680
		30 min				60 min	−1.783 ± 1.29	−1.384	0.366
		60 min			30 min	60 min	−0.870 ± 1.03	−0.846	0.679
	DBP	0 min	0.206	0.815	0 min	30 min	0.231 ± 0.915	0.252	0.966
		30 min				60 min	−0.346 ± 1.001	−0.346	0.936
		60 min			30 min	60 min	−0.577 ± 0.785	−0.735	0.745
RA	SBP	0 min	1.63	0.210	0 min	30 min	0.476 ± 0.953	0.500	0.872
		30 min				60 min	−1.381 ± 1.233	−1.120	0.513
		60 min			30 min	60 min	−1.857 ± 1.003	−1.851	0.179
	DBP	0 min	1.14	0.328	0 min	30 min	−0.692 ± 0.815	−0.850	0.676
		30 min				60 min	−1.154 ± 0.817	−1.413	0.350
		60 min			30 min	60 min	−0.462 ± 0.666	−0.693	0.770

Abbreviations: CCA, Common carotid artery; DBP, Diastolic blood pressure; F, F statistic; RA, Radial artery; SBP, Systolic blood pressure; SE, Standard error; t, t statistic.
